# Circulating microRNAs in Breast Milk and Their Potential Impact on the Infant

**DOI:** 10.3390/nu12103066

**Published:** 2020-10-08

**Authors:** Elena Carrillo-Lozano, Fernando Sebastián-Valles, Carolina Knott-Torcal

**Affiliations:** Department of Endocrinology and Nutrition, Hospital Universitario de la Princesa, Instituto de Investigación Princesa, Universidad Autónoma de Madrid, 28006 Madrid, Spain; e.carrillolozano@gmail.com (E.C.-L.); fer.sebas.valles@gmail.com (F.S.-V.)

**Keywords:** miRNAs, breast milk, exosomes, breastfeeding, gene expression

## Abstract

MicroRNAs (MiRNAs) are small RNA molecules that can exert regulatory functions in gene expression. MiRNAs have been identified in diverse tissues and biological fluids, both in the context of health and disease. Breastfeeding has been widely recognized for its superior nutritional benefits; however, a number of bioactive compounds have been found to transcend these well-documented nutritional contributions. Breast milk was identified as a rich source of miRNAs. There has been increasing interest about their potential ability to transfer to the offspring as well as what their specific involvement is within the benefits of breast milk in the infant. In comparison to breast milk, formula milk lacks many of the benefits of breastfeeding, which is thought to be a result of the absence of some of these bioactive compounds. In recent years, the miRNA profile of breast milk has been widely studied, along with the possible transfer mechanisms throughout the infant’s digestive tract and the role of miRNA-modulated genes and their potential protective and regulatory functions. Nonetheless, to date, the current evidence is not consistent, as many methodological limitations have been identified; hence, discrepancies exits about the biological functions of miRNAs. Further research is needed to provide thorough knowledge in this field.

## 1. Introduction

MicroRNAs (also called miRNAs) are short, non-coding RNA sequences (approximately 22 nucleotides), which act as post-transcriptional regulators of gene expression. The biogenesis of miRNA is initiated by the enzyme RNA polymerase II, producing precursor miRNA molecules (primary miRNAs or pri-miRNAs) [1]. These molecules are then subjected to modifications by diverse proteins from the RNase III family. In the nucleus, the enzymatic complex called Drosha-DGCR8 (DiGeorge Syndrome critical region gene 8 protein) processes the pri-miRNAs, producing a shorter molecule called pre-miRNA. In the cytoplasm, Dicer is the ribonuclease responsible for the creation of mature miRNAs from pre-miRNA. Mature miRNA, which is bonded to an Argonaute protein (AGO), binds to the RNA-induced silencing complex (RISC), where a single strand is retained to perform its function [2]. The mechanism of action of the miRNA is based on the interaction of complementary sequences of miRNA in the 3′-untranslated region (3′-UTR), causing its degradation or inhibiting the translation into proteins [1]. Precisely, whereas AGO2 protein is able to degrade miRNAs, AGO1, 3, and 4 can inhibit protein translation [3].

MiRNAs are known to be ubiquitous, as they can be found in different tissues, [4] cells, and biological fluids such as blood, plasma, saliva, urine, tears, sperm, or human milk amongst others [5,6]. It appears that a great part of them can circulate bonded to AGO proteins [7]. Although the majority of miRNAs have similar binding affinity to AGO1 and AGO2, some miRNA species may preferably bind to one or another. This suggests a potential contribution of different tissues to the extracellular miRNA pool [3]. The sensitivity of its determination is incremented when using RT-PCR amplification [8]. There are diverse bioinformatics tools to help predict the potential functions of the different miRNAs that have been studied [9]. Each miRNA has been attributed to have an effect on hundreds of genes, regulating up to 60% of the genes coding for proteins [10]. Different miRNAs have been related to physiological processes such as cell growth or immunity [11]. They have also been involved in different conditions, including cardiovascular disease, autoimmune diseases, or cancer [9]. In this context, there is an increasing interest in their application as non-invasive biomarkers [5,9] as well as in their potential therapeutic uses [12].

In addition to the endogenous miRNAs, the potential impact of the exogenous ones has become even more significant in recent years [13,14]. Emerging evidence points out that miRNAs contained in different dietary sources can play a key role in epigenetic regulation processes and intercellular communication [15,16,17].

Breastfeeding has demonstrated numerous benefits for both the mother and the infant compared to formula feeding. Thus, the World Health Organization (WHO) recommends exclusive breastfeeding during the first 6 months and as a complement up until 2 years [18]. Several miRNAs have been identified in human breast milk [19,20], with potential implications to regulate genes with different functions. The aim of this review is to summarize the evidence about miRNAs’ characteristics, potential functions, as well as different factors involved in their regulation.

### Components of Human Breast Milk: Nutrients and miRNAs

Regarding macronutrients, lactose is the most abundant carbohydrate in human breast milk [21]. Furthermore, oligosaccharides can also be found in human milk, which play an important role as prebiotics to help develop the gut microbiota in the infant’s gastrointestinal tract. Lipids, which are present as an emulsion, constitute the main source of energy. Triacylglycerides constitute approximately 98% of the lipid fraction. Other lipids such as phospholipids, monoacylglycerides, diacylglycerides, or free fatty acids mainly make up the remaining fraction. These components are usually encapsulated into milk fat lipid globules [22]. Regarding proteins, human breast milk contains more than 400 different ones, which can be divided into three main groups: casein, whey, and mucin proteins. However, the proteins’ ratio varies and gradually changes over time. For example, the whey/casein ratio changes as the lactation progresses, from 90/10 in the colostrum from the first days to 60/40 in mature milk. It also contains non-protein nitrogen, which makes up to 25% of the total nitrogen present in milk. Additionally, human breast milk usually provides suitable amounts of micronutrients for the infant needs, except for vitamins K and D, which are recommended to be routinely supplemented in exclusively breastfed infants [23].

In recent years, there has been an increasing interest in the composition of human breast milk, which has gradually changed from the nutritional components to its bioactive compounds [23], such as miRNAs, maternal cells, and antibodies, especially secretory immunoglobulins (SIgA or SIgG), whose concentration is particularly high in early lactation [22]. Breast milk has been classified as one of the biological fluids with a richer content in miRNAs, with approximately 1400 different miRNAs identified. These miRNAs have been found in the different fractions of breast milk (cells, lipids, and the skim milk), as either free molecules or packaged in vesicles [14], and it seems that they might be synthesized in the mammary epithelial cells. Although miRNAs had been previously studied in the skim fraction of the milk, recent studies in the lipid and cell fractions have shown a greater amount and diversity than that in the skim one [24].

Many similarities have been found between the miRNA profile of human breast milk and that of the milk of other mammalian species [17]. A study that compared milk from different mammals found that approximately 91–92% of the miRNA profile expressed in human breast milk was also expressed in bovine and goat milk. What is more, 89% and 83% of the miRNAs expressed in bovine and goat milk, respectively, were also found in human breast milk. In addition, the miRNAs more highly expressed in the different milk fractions were quite similar between the species, particularly MiR-148a-3p, which is one of the most highly expressed in both the skim and lipid fractions, even after pasteurization [25]. This same study also analyzed the availability of MiR-148a-3p in different commercial infant formulas, finding significantly lower levels of this miRNA compared to that in breast milk. Moreover, another study analyzed the content of miRNAs in purified milk-derived extracellular vesicles from human and porcine milk [26] and then compared these results with data from previous studies in human, porcine, cow, and panda milk. It was demonstrated that a large number of the top 20 miRNAs more abundantly present in breast milk were also common between the different mammalian species.

The composition as well as the expression of miRNAs in breast milk can fluctuate for many reasons, and one of the factors involved in these variations seems to be the process of feeding. Alsaweed et al. [24] conducted a study to analyze milk collected from 16 women during the second month of exclusive breastfeeding, both before and immediately after the feeding. The vast majority of the known miRNAs were found before and after the feeding although some feeding-specific miRNA were also identified. On the one hand, 159 and 180 feeding-specific miRNAs were exclusively identified in pre-feed and post-feed samples respectively, which means that none of the pre-feed milk-specific miRNAs were identified in any post-feed samples and vice versa. These feeding-specific miRNAs were expressed at low concentrations. On the other hand, most of the top 10 most highly expressed known miRNAs were similarly expressed in both pre- and post- feeding within an infant/mother dyad. Additionally, nearly all the known miRNAs most highly expressed in the cell fraction of the human breast milk were common in all the mothers who participated in the study. Individual variations were found within mother/infant dyads, especially regarding novel species of miRNAs, what suggests that there is a component of regulation mediated by miRNAs that is dyad specific.

Apart from the aforementioned feeding-related variations, changes have also been described in the expression and content of miRNAs as the lactation progresses. Alsaweed et al. [27] carried out a study in the cell and lipid fractions of the human breast milk, where they collected post-feed samples from 10 lactating women in months 2, 4, and 6. In these samples, 1159 mature, known miRNAs were found, and approximately two thirds of the known miRNAs common in both fractions were very similarly expressed. Likewise, out of the 5167 novel miRNAs that were initially predicted, just 235 were high-confidence miRNAs. They concluded that the profile of novel miRNAs was quite different between both fractions, with the cell fraction being richer in novel miRNA species than the lipid fraction. In addition, the vast majority of novel miRNAs were mother-specific. Regarding differences between lactating periods (months 2, 4, and 6), most of the known miRNAs were very similarly expressed when considering both fractions together (cell and lipids). Furthermore, of the differentially expressed miRNAs, known miRNAs were upregulated at month 4 compared to those at months 2 and 6. Whereas the majority of novel miRNAs were expressed at low levels and showed great variation intra- and intergroup during the first six months of lactation in both milk fractions. Additionally, some situations such as stress, immune dysfunction, or mastitis have been demonstrated to influence the miRNA expression pattern.

Finally, similarly to miRNA, it has been recently described that other small RNA species circulating in human breast milk, such as circular RNAs (circRNAs), transfer RNAs (tRNAs), small nuclear RNAs (snRNAs), small nucleolar RNAs (snoRNAs), long non-coding RNAs (lncRNAs), and piwi-interacting RNAs (piRNAs) [28,29], have a differently expressed profile in plasma compared to that in human milk.

## 2. Transport and Absorption of Breast-Milk-Derived miRNAs

### 2.1. Mechanisms of miRNA to Resist Degradation

In order to perform any of their functions, miRNAs ingested through the human breast milk must overcome a number of obstacles. The gastrointestinal tract is a tough environment because of factors such as pH, RNases, the gut microbiota, or the intercellular junctions between enterocytes. After passing from the digestive tract to the circulation, the circulating enzymes, the immune system, the vascular endothelium, and the membrane itself involve potential hazards for the integrity of the miRNA [14,17].

The ability of the miRNAs to overcome all these barriers and get to their target in a specific concentration has been questioned [30]. Nonetheless, recent studies have highlighted that exosomes play a very important role. Exosomes are small, extracellular vesicles of approximately 30–100 nm, which are produced by a variety of cells and are capable of transferring information to the receptor cells through their content [31]. Additionally, exosomes have been isolated in human breast milk and it has been proven that they contain small RNA molecules [32,33] as well as membrane and cytosolic proteins involved in cell signaling [34]. The membrane of the exosome can act as a barrier that protects its content against potential hazards [19]. In a study where bovine milk was placed in a lower pH environment to simulate the conditions of the gastrointestinal tract, the change in acidity did not affect the efficiency or the quality of the miRNA present in the bovine-milk-derived exosomes [35]. Moreover, miRNAs from pasteurized cow milk have been demonstrated to be stable despite the degradative conditions of the pasteurization process [20,36].

In addition to that, Golan et al. [25] observed that the expression of miRNAs in the different fractions of the milk remains similar even after the pasteurization process. They suggest that whereas exosomes appear to protect miRNA in the skim milk, this protective effect in the lipid fraction might be due to the exosomes as well, or even because of fat globules or proteins such as Argonaute-2. Lin et al. [37] also proposed that milk-derived miRNA may circulate in plasma joined to proteins, as occurs with some endogenous known miRNA [7,38,39]. Another recent study from Smyczynska et al. suggests that from all the pasteurization techniques, the High-Pressure Processing method interfere less with miRNA functionality compared to the Holder method [40].

### 2.2. Presence of miRNA in the Digestive System, Absorption, and Biological Effect

Whereas the presence of miRNA in breast milk is an acknowledged fact, there is still a lot of controversy about the potential absorption and effects in the offspring to date. The dissenting evidence has given rise to the advocacy of two different theories regarding the effects of miRNAs. On the one hand, some authors support a functional hypothesis, which stands for the transfer of breast milk miRNAs and their consequent epigenetic regulation in the offspring. However, other authors support a nutritional hypothesis, which considers that miRNAs cannot reach the systemic circulation to exert their regulatory role and, therefore, restrict their role to mere nutrients [41].

A recent pilot study was conducted to analyze the miRNA profiling in gastric content from both breast-fed and formula-fed infants [11]. In this study, they analyzed the gastric content of infants at autopsy and demonstrated the presence of miRNAs in gastric content. What is more interesting about these findings is the fact that the samples were isolated between 1 month and 2 years after their death. Additionally, they found differences in the miRNA content regarding the type of feeding (breastfed or formula fed) and, in this framework, they propose miR-151a and miR-186 as potential breast milk biomarkers.

In regard to digestion and absorption, Liao et al. [42] carried out an in vitro experiment simulating digestion in order to study the miRNA behavior throughout this process. They proved that miRNAS in human milk exosomes (both digested and undigested) were taken up by the cripta-like intestinal cells, which elucidates how miRNA respond to the digestion process and is able to survive. Apart from that, Baier et al. [43] studied the gene expression and milk miRNA depletion in human cell cultures and mice, respectively. They examined the expression pattern of miRNAs after consumption of different doses of cow milk in healthy adults and found a significant increase of mi-R-29b in serum after ingestion, which returned to the initial levels after a dose-dependent period of time. After analyzing peripheral blood mononuclear cells (PBMCs), they also objectified an increase in the expression of miR-200 and miR-29b. Similarly, they also found a significant increase in the expression of the gene *RUNX2* (runt-related transcription factor 2), which is a known target for miR-29b, and further observed that a depletion of exogenous intake of miRNA cannot be compensated by endogenous synthesis. The authors concluded that milk-derived miRNAs can be efficiently absorbed and regulate human gene expression, all this being dose-dependent.

Lin et al. [37] analyzed the miRNA profile in piglet serum after oral administration of bovine and porcine milk and studied the differences in miRNA expression profiles both in in vivo and in vitro experiments. Milk-derived miRNAs were found in the piglets’ serum. Similarly, the results of the incubation of bovine and porcine milk exosomes and supernatants showed an increase in the levels of miRNAs in IPEC-J2 cells (Intestinal Porcine Epithelial Cells from jejunum) proportional to the miRNAs’ levels in the milk, which means that a higher content of miRNAs in the milk samples resulted in a higher content of miRNAs in IPEC-J2 cells. These data are consistent with the hypothesis that exogenous miRNAs can be absorbed through the digestive tract.

According to an experiment, conducted by Kusuma et al. [44], in which human umbilical vein endothelium cells (HUVEC) were used, they concluded that human vascular endothelial cells can transport milk-derived exosomes by endocytosis. In this study, they also observed the ability of milk exosomes to transfer across the endothelial layer as well as the compatibility of milk exosomes’ surface proteins with the proteins on the surface of human vascular endothelial cells.

Regarding the availability of milk miRNAs in tissues, radiolabeled exosomes of cow milk containing miRNAs were identified in murine models in an experiment carried out by Manca et al. [45]. Their presence was confirmed at different levels such as intestinal mucosal barrier, spleen, liver, heart, and brain. Likewise, Kosaka et al. [46] demonstrated the uptake of human-milk-derived exosomes in an experiment conducted in samples of human cell lines.

Contrary to the aforementioned results of different studies, Auerbach et al. [47] did not find any evidence of the presence of milk miRNAs either in plasma or in peripheral blood mononuclear cells (PBMCs) despite analyzing the same samples as Baier et al. [43]. However, the authors considered the different results could have been due to questionable quality and stability of the previously frozen samples, the use of different analysis techniques, and the lack of a consensus on cut-off values to confirm to presence of miRNA. Similarly, Laubier et al. [48] demonstrated a high concentration of miR-30b in milk in a transgenic mouse model, and although it was also found in gastric content, they could not objectify its presence in blood or any other tissues. Another study conducted in KO mice (knockout mice) which assessed the absorption, uptake, distribution as well as potential effects of the miRNA from the mother, did not find a significant presence of exogenous miRNA in the offspring [30].

Recent studies from Wang et al. [15] have shifted the emphasis of this controversy towards the nutritional hypothesis. The authors were able to confirm with RNase H2-dependent PCR (rh-PCR) the presence in human plasma of bovine-milk-specific miRNAs after its intake, particularly miRNA-21 and miRNA-30a. Similarly, they identified other technical factors that may damage the stability of these miRNAs, such as sample collecting in heparin tubes, hemolysis, and storage thermal conditions. Likewise, they suggested new experimental foundations, which are necessary for reliable measures of miRNA, and questioned previous works in which some of these factors may have contributed to the lack of proof of miRNA absorption in bovine milk.

Furthermore, in the pharmacological field, some investigations enhance the effective absorption and biological role of miRNAs [12,16,49]. In this regard, bovine-milk-derived exosomes are being looked into as potential carriers for the oral administration of current intravenously administered drugs as well as potential agents for cancer treatment, having obtained promising results in murine models and preclinical studies.

## 3. Potential Effects of miRNA Found in Human Breast Milk

Diverse functions of the miRNAs isolated from breast milk have been described, although with different levels of evidence. Melnik et al. carried out an extensive review in which they described that the suppression of DNMT (DNA methyltransferase) mediated by miRNAs such as miR-148a, results in a hypomethylation and, consequently, in an activation of genes with different functions. Experimental evidence supports that milk exosome-derived MiRNAs, such as miR-148a, induce proliferation and protein expression in normal but not in tumor cells [50]. The main implications in the development would be in the metabolism (*INS, IGF1, CAV1*), immunity (*FOXP3, NRA4*), adipogenesis (*FTO, FABP4, CAV1, PPARG2, SREBP1, LPL*), myogenic programming (*NR4A3*), osteogenesis (*NRF2*) and epidermis (*NRF2*) [41]. A summary of the possible roles of some miRNAs will be listed below.

Many studies have assessed the role of miRNA in immunity and linked its presence to the regulation of immune responses. High levels of miRNAs with immunological functions in milk-derived exosomes support the potential role of these miRNAs in the development, proliferation, maturation, and regulation of the immune system, as breastfeeding has been described to have [2]. Kosaka et al. [46] observed that in breast milk from the first six months of lactation there was a high content of miR-181 and miR-155, involved in the differentiation of B cells. Other miRNAs, such as miR-17 and miR-92, which are also present in high levels in breast milk, have been associated with the regulation of monocyte development as well as the differentiation and maturation of B and T cells [51]. Lastly, miR-223 has been associated with a proliferation of granulocytes [52]. However, other breast milk miRNAs have also been linked to having an immunomodulatory effect. MiR-150, which is supposed to occur at low concentrations, acts as a suppressor of B cells [53]. In a study of Zhou et al. [19] regarding miRNAs in breast milk-derived exosomes, 4 of them were associated with immune functions, namely, miR-148a-3p, miR-30b-5p, miR-182-5p, and miR-200a-3p. These results showed a complex relationship in their regulation, even with antagonist functions, depending on the target of action of each miRNA. For instance, whereas miR-30b-5p can induce immunosuppression and reduce the activation of several immune cells [54], miR-182-5p can induce T cell-mediated immune responses [55].

Regarding mechanisms of allergies, epidemiological evidence shows a protective effect of breast milk against the development of atopy [56]. Precisely, bovine and human milk are very rich in exosomal miR-155 [36], which has been attributed a role in this context. Kohlhaas et al. observed a reduced number of T regulatory cells (Tregs) both in the thymus and peripheral tissues in murine models who were deficient in miR-155, what suggests that this miRNA might be necessary for the development of Tregs [57]. A possible explanation might be that suppression of miR-155 may result in limited signaling of IL-2/STAT5 (Interleukine-2/ Signal Transducer and Activator of Transcription 5), which actually reduces the supply of Tregs [58]. Additionally, Admyre et al. incubated human peripheral blood mononuclear cells (PBMCs) with isolated human milk exosomes for regulatory T cell analysis, which interestingly showed that milk exosomes induced a dose-dependent increase of the number of Foxp3+ CD4+ CD25+ T regulatory cells (Tregs) [32].

As suggested by Melnik et al. [59], the current evidence supports the idea that milk-derived exosomal miRNAs might be involved in the development of Tregs and provide a potential cellular signaling network that can adequately control the maturation of Tregs and, consequently, prevent atopic allergy, in the context of raw cow’s milk consumption. This is because it is known that raw milk contains the highest amounts of miRNAs compared to pasteurized milk in which miRNA levels are lower. Similarly, boiling of milk can degrade bioactive milk miRNAs and destroy their biological activity [19], by causing the potential atopy-protective effects to decline [60]. It is believed that the mechanism for this could be a disruption of the lipid layer of milk exosomes, which would result in miRNA exposure and subsequent degradation by RNase.

The absence of miRNA-155 in infant formula could lead to inadequate development of Tregs. In this context, the addition of miR-155-enriched exosomes to infant formula has been suggested to prevent the onset of atopic diseases [59].

Moreover, recent concepts link milk-derived miRNA-148a to pancreatic beta-cell differentiation, which highlights a potential breastfeeding-protective role against type 2 diabetes mellitus development [61].

Regarding lipid metabolism, miR-125a-5p can be abundantly found in human and other mammals’ breast milk and it is known to regulate oxysterol-binding protein-related protein (ORP) 9, which is involved in diverse processes of lipid metabolism [62] such as the induction of lipid absorption by macrophages. Furthermore, miR-193b and miR-365 have been attributed an upregulation of the differentiation process of the brown adipose tissue through an improvement of the expression of Runt-related transcription factor 1, translocated to 1 (*RUNX1T1*) [63].

In relation to neurodevelopment, many miRNAs present in breast milk have been linked to synaptic plasticity, cognitive capacity, and neurological disorders in murine models [64]. Lönnerdal et al. [65] examined the miRNAs with possible effects in the development of the synapsis in mammals and compared them to the ones found in breast milk. Interestingly, approximately half of them were present in the top 288 of human breast-milk-derived exosomal miRNAs. The presence of these miRNAs suggests that early brain development may benefit from the miRNA effects in this regard.

In addition to the biological functions that miRNAs exert in the newborn, it has been proven that miRNAs could also be involved in regulating lactation, milk production, and its composition. In a study conducted by Alsaweed et al. [24], they found that eight of the most highly expressed miRNAs in the cell fraction of the human breast milk appeared to play an important role in producing and maintaining triacylglycerol levels. They further observed that six of the most highly expressed miRNAs in the cell fraction were associated with the regulation of different genes involved in fatty acid biosynthesis, including palmitic acid, oleic acid, and stearic acid. Lactose synthesis, which specifically takes places in the mammary gland, was also found to be regulated by four of the most highly expressed miRNAs, mainly by miR-182-5p and let-7f-5p, which regulate UDP-glucose transporter (*SLC2A3*) and UDP-galactose transporter (*SL35A2*). Furthermore, epigenetic regulation of DNMT (DNA methyltransferases) has been proposed as an important mechanism to promote efficient lactation and a suitable synthesis of proteins and lipids in the mammary gland [66]. Apart from that, studies in animal models have identified miRNAs that are also involved in their milk’s composition, such as miR-103 and miR-145, which are associated with lipid synthesis in goat and mouse milk, respectively [67].

Regarding miRNA content, it has been suggested that it varies as the lactation progresses in order to adapt to the infant needs. Alsaweed et al. [24] claim that the miRNA composition in both fractions of the human breast milk (cell and lipid) does not change much in the first 6 months of lactation, although it seems to be particularly altered in month 4 of lactation, when a more pronounced miRNA upregulation occurs. This may indicate the remodeling of the mammary gland according to a change in the infant feeding patterns after exclusive breastfeeding.

Some of the aforementioned miRNAs and their functions have been gathered and summarized in Table 1.

## 4. Evidence about the Influence of Milk Exosome-Derived miRNAs in Intestinal Maturation and Inflammation: Inflammatory Bowel Disease and Necrotizing Enterocolitis

In a recent study in murine models carried out by Reif et al. [68], they assessed the therapeutic effect of human and cow breast milk in a model of dextran sulfate sodium–induced colitis. The authors observed that both human- and bovine-milk-derived exosomes reduced the level of histopathological scores and colonic shortening, as well as interleukin 6 (IL-6) and TNF (tumor necrosis factor) expression. Furthermore, highly expressed miRNAs were found in a greater proportion in milk-derived exosomes, such as miRNA-375, let-7a, miRNA-148, and miRNA-320, in the colon of mice treated with milk-derived exosomes. They further observed a downregulation in DNA methyltransferase 1 and 3 (DNMT1 and DNMT3) as well as increased levels of transforming growth factor- beta (TGF-β) in the colon of mice treated with milk-derived exosomes.

Another study [69] assessed the effect of exosomes from commercial cow’s milk in dextran sulfate sodium–induced colitis in a murine model. They noticed an improvement in several features of the dextran sulfate sodium–induced colitis, a modulation of the gut microbiota as well as a restored intestinal permeability and replenished mucin secretion. They also noticed an effect in the modulation of the innate immune response and a decrease in inflammation through the downregulation of miRNAs associated with colitis, especially miR-125b, which has been related to a higher expression of the NF-κB (Nuclear Factor Kappa B) inhibitor *TNFAIP3* (TNF Alpha Induced Protein 3).

The level of inflammation of ulcerative colitis might also be modulated by exosomes isolated from commercial cow’s milk. In a study in a murine model, [70] milk-derived exosomes prevented the onset of the severe ulcerative phenotype, therefore improving colitis macroscopic damage scores as well as other surrogate parameters such as weight, colonic length, or stool volume.

Furthermore, Xie et al. [71] carried out an in vivo study regarding the potential role of porcine-milk-derived exosomes in mice that had been treated with deoxynivalenol toxin (DON), which can cause severe damage to the intestinal mucosa. The authors suggest that milk-derived exosomes might revert the induced inhibition of the cell proliferation and decrease the deoxynivalenol-induced apoptosis. This can consequently upregulate some miRNAs such as miR-181a, miR-30c, miR-365-5p, and miR-769-3p in IPEC-J2 cells, which downregulate the expression of target genes in the p53 pathway.

Milk-derived exosomes have also been proposed as future potential candidates in the treatment of necrotizing enterocolitis [72]. Several studies conducted in murine models have demonstrated a decrease in the incidence of necrotizing enterocolitis in rat offspring when human-milk-derived exosomes were administered orally compared to intraperitoneal administration [42].

In intestinal cell culture models in rats (IEC-6) and humans (FHS74), human-milk-derived exosomes have shown pro-proliferation and anti-apoptotic effects, which suggests that the therapeutic properties are mediated by a protective effect over the intestinal epithelial cells [73]. They also seem to reduce the severity of necrotizing enterocolitis by altering the expression of multiple proteins. An example of this could be a decrease in myeloperoxidase, a pro-inflammatory molecule released by neutrophils, or an increase in *MUC2* (Mucin 2) and *GRP94* (Glucose-Regulated Protein 94), which are expressed by calceiform cells and are the main components of the intact gut barrier [74].

## 5. Influence of the Diet and Other Maternal Factors in the Composition of miRNAs in Breast Milk

Different features and dietary patterns have been examined as potential factors involved in the nutritional content of breast milk [75]. Evidence in this topic is very limited, although it is believed that the fatty acid profile and some micronutrients are more likely to vary [75,76]. Additionally, maternal obesity has been linked to changes in the nutritional composition and regulatory factors in breast milk [77,78]. Apart from that, maternal features are also involved in the content of expressed miRNA in breast milk.

An obesogenic dietary pattern, or so-called cafeteria diet, seems to lead to variations in specific miRNA levels in breast milk, according a study conducted in nursing rats [79]. Those following the cafeteria diet presented higher concentrations of miR-222 and lower ones of miR-200 and miR-26 compared to controls. In addition, offspring of the cafeteria group showed a lower expression of *CDKN1C* (Cyclin Dependent Kinase Inhibitor 1C) in liver than controls, which is known to be involved in growth as well as a validated target gene of miR-22. Additionally, the same author demonstrated that this dietary pattern can alter the content of bioactive proteins in milk, causing increased levels of leptin and adiponectin and decreased levels of irisin, with a consequent impact on the offspring’s metabolism [80]. Another study [81] objectified an increase in the expression of novel miRNAs in milk fat globules, in particular, miR-67 and miR-27, in women following a high-fat diet, compared to those following a high-carbohydrate diet (both isocaloric-isonitrogenous diets).

The possible effects of maternal excessive body weight have been assessed in two different studies in humans. In one of them [82], adipogenesis-related miRNAs, including let-7a, miR-30B, and miR-378, were downregulated in relation to higher body max index (BMI) values. Zamanillo et al. [83] identified an alteration in breast milk miRNAs associated with leptin and adiponectin function in women with BMI values >25 kg/m^2^. This coincided with the fact that none of those hormones decreased throughout the lactation in women with obesity or overweight. Additionally, they further observed an altered relationship between miRNAs and other features, such as milk lipid composition and offspring’s BMI at two years. Those miRNAs with an altered expression profile in relation to excessive body weight (miR-let-7a, miR-103, miR-222, miR-17, miR-146b, miR-30) were linked to different process such as cellular and brain development, as well as miRNA transport and release.

Mirza [84] found a disrupted pattern in the concentrations of breast milk exosomal miRNAs in mothers with type 1 diabetes mellitus, compared to healthy mothers. Whereas has-miR-4497, has-miR-1246, has-miR-133a-3p, has-miR-3178, has-miR-1290, and has-miR-320d were upregulated, has-miR-518e-3p, has-miR-29-3p, and has-miR-200c-5p were downregulated. Some of these variations were associated with genes involved in the immune response. These changes were not correlated to BMI values or metabolic control of their diabetes. The authors highlighted that the impact of those findings was not established and that they did not mean to justify a recommendation against breastfeeding in mothers with type 1 diabetes mellitus.

Ultimately, it seems that maternal features related to diet, metabolic state, or the presence of diseases might alter the miRNA expression profile in breast milk. However, the potential implications in the regulation of the growth and development, immunity as well as in the metabolism require further research.

## 6. Potential Limitations and Controversies

Even though human breast milk has been recognized as a rich source of miRNAs, the implications of these miRNAs in the infant and the mother have not been fully defined, as lots of discrepancies can be found between studies. Additionally, the sample size in the vast majority of the studies is generally limited, especially those in humans.

Differences in the sample treatment, for example, milk removal, storage and preservation as well as processing are a potential source of divergence in this field. Many discrepancies have also been found depending on the milk fraction that was analyzed, skim, cell, or lipids, according to the aforementioned literature.

Additionally, studies in breast milk have used different techniques to detect miRNAs, from PCR, qPCR, and microarrays to next-generation sequencing. The fact of using one or another might influence the results. Apart from that, it is also important to highlight that there is not a universally established cut-off value to consider the results as clinically relevant.

It is also important to distinguish the concepts of exosomes and other extracellular vesicles, which is necessary to interpret data appropriately. From a methodological point of view, laboratory techniques to properly isolate exosomes or their markers regarding some of their characteristics, such as size or density, are also required [34]. Another great challenge is the difficulty to obtain sufficient amounts of small RNA molecules from exosomes in order to establish a representative profile [85].

An additional source of error might be the difficulty to differentiate between endogenous miRNAs and the exogenous ones transferred through breast milk. Conducting studies using radiolabeled exosomes [41] or the use of methods such as rhPCR (RNase H2-dependent polymerase chain reaction) [86] could facilitate that process.

Lastly, the causal relationship between the presence of miRNAs as well as their absorption and biological action has not been clearly established, to date, and further research is needed. The greatest evidence in this regard is limited to results from animal models and in vitro experiments and, therefore, it is challenging to extrapolate the results to humans. For instance, it has been suggested that some animal models such as KO mice could not be the most appropriate ones to study physiology of maternal–offspring transfer of miRNAs [41].

## 7. Conclusions

The benefits of breastfeeding are widely acknowledged. Emerging data clearly shows that far from just being a nutritional product, breast milk can act as a complex cell signaling system and is rich in bioactive compounds involved in the correct growth and development of the infant. Particularly, miRNAs exert a recognized role in the regulation of gene expression. The recognition of breast milk as a source of miRNAs has aroused an increasing interest about their potential transfer to the offspring as well as breast milk involvement in the aforementioned regulatory functions of the gene expression. Accumulating translational evidence in human and animal studies supports the functional concept of milk exosomal miRNAs as signalosomes that reach the infant and the human consumer of bovine milk. The current evidence is genuinely favorable regarding that role, although further research in this topic is needed. Ultimately, future works with larger sample sizes, focusing on humans, and using standardized methodology will elucidate and broaden our knowledge in this field.

## Figures and Tables

**Table 1:** Functions of micro RNAs (miRNAs).

miRNA	Functions	Reference
miR-181, miR-155	Differentiation of B cells.	[46]
miR17, miR92	Regulation of monocyte development. Differentiation and maturation of T and B cells.	[51]
miR-223	Proliferation of granulocytes.	[52]
miR-150	Suppression of B cells.	[53]
miR-200a-3p	Involved in Hodgkin lymphoma and oral cancers.	[19]
miR-148a-3p	Negative regulation of immunity.Different roles in metabolism and development.	[19,41]
miR-30b-5p	Immunosuppression.	[54]
miR-182-5p	T-cell activation. Lactose synthesis in mammary gland. Regulation of UDP-glucose transporter (SLC2A3).	[56,24]
miR-155	Development of T-reg cells (TREG).	[58]
miR-125-5p	Regulation of oxysterol-binding protein-related protein (ORP) 9, involved in lipid metabolism.	[62]
miR-193b, 3iR-365	Differentiation of brown adipose tissue.	[63]
let-7f-5p	Lactose synthesis in mammary gland. Regulation of UDP-galactose transporter (*SLC35A2*).	[24]
miR-103, miR-145	Lipid synthesis in mammary gland.	[67]

UDP: Uridine Diphosphate-glucose transporter.
